# The Melatonin Treatment Improves the Ovarian Responses After Superstimulation in Thai-Holstein Crossbreeds Under Heat Stress Conditions

**DOI:** 10.3389/fvets.2022.888039

**Published:** 2022-04-28

**Authors:** Ruthaiporn Ratchamak, Pachara Thananurak, Wuttigrai Boonkum, Yoswaris Semaming, Vibuntita Chankitisakul

**Affiliations:** ^1^Department of Animal Science, Faculty of Agriculture, Khon Kaen University, Khon Kaen, Thailand; ^2^Network Center for Animal Breeding and Omics Research, Faculty of Agriculture, Khon Kaen University, Khon Kaen, Thailand; ^3^Program in Veterinary Technology, Faculty of Technology, Udon Thani Rajabhat University, Udon Thani, Thailand

**Keywords:** dairy cows, follicular development, oxidative stress, ROS, superstimulation, THI (temperature humidity index)

## Abstract

The effect of heat stress with melatonin treatment on the superovulatory responses and embryo characteristics in Thai-Holstein crossbreeds under heat stress conditions was examined. Six non-lactating cows (replication = 4; *n* = 24) were assigned to one of 2 treatments in double cross-over design. All cows were superstimulated with traditional treatment. Melatonin group (*n* = 12): cows received intramuscularly injection 18 mg/50 kg. simultaneously with GnRH injection, while those in the control group (*n* = 12) received none. Bloods samples were taken to determine lipid peroxidation (MDA) and the activity of the antioxidant enzymes (superoxide dismutase; SOD). The experiment was conducted from April to September, which determined severe heat stress (the mean temperature-humidity index above 77). The results revealed that numbers of large follicles and corpora lutea were higher in the melatonin group than in the control group (*p* < 0.01). Numbers of recovered ova/embryos, fertilized ova, and transferable embryos were higher in the melatonin group (*p* < 0.01); meanwhile, more degenerated embryos were found in the control group (*p* < 0.01). Increased activity of the antioxidant enzymes SOD after melatonin administration decreased MDA levels (*p* < 0.05). In summary, melatonin administration benefited the ovarian response and embryo quality in superstimulated Thai-Holstein crossbreed affected by heat stress.

## Introduction

Embryo transfer has been used rather than artificial insemination to enhance pregnancy rates throughout the summer season as the transferrable blastocysts have greater heat resistance than *in-vivo* derived embryos (1). However, the good quality of transferrable embryos should be collected from non-heat stressed cattle (2). Our previous study reported that heat load during the superovulatory treatment affected the superovulatory response (3). The reduction in large follicles and corpora lutea was observed at a temperature-humidity index (THI) of 72. Significant declivity in the embryo development was evidently after THI of 77.

Heat stress seems to modify folliculogenesis efficiency and adversely affects follicle quality (4). In a study of oocyte culture *in vitro*, heat shock of the oocyte in thermal stress affected morphological changes in the cells which are oxidative stress, nuclear fragmentation, and mitochondrial impairment (5, 6). Moreover, heat stress affects cell function and breaks the DNA or organ functions by inducing oxidative stress (reactive oxygen species; ROS) (7). This induces DNA cell damage, leads to apoptosis lipid peroxidation, and breaks the mitochondrial function, resulting in cell death eventually (8, 9). Besides oocyte maturation failure, Orgal et al. (10) report that heat shock during *in vitro* fertilization reduces the fertilized rate and embryonic development. Therefore, heat stress is an important factor in increasing oxidative stress and is negatively associated with oocyte quality, embryo quality, and IVF outcomes (10, 11). Hence, increasing the cell's resistance to ROS by adding antioxidants would be an alternative way to improve the efficiency of superovulatory response.

Melatonin (N-acetyl-5-methoxytryptamine) is an endogenous hormone that is produced by the pineal gland. It is both fat-soluble and water-soluble to quickly pass-through organic membranes (12). Furthermore, intracellular organelles in the cell nucleus and mitochondria could be protected from oxidative damage directly at the sites where such damage occurs (13, 14). During the past decade, the beneficial effect of melatonin supplementation as a powerful agent against ROS from oxidative stress and degeneration has been reported during *in vitro* embryo production to improve oocyte maturation, fertilization rate, and embryo development by supplementing in the culture medium such as in buffalo (15), and bovine (16). Meanwhile, almost studies of *in vivo* production are used to promote reproductive performances in seasonal breeding animals such as sheep (17, 18) and deer (19, 20), in which melatonin regulates the cycling of reproductive activity. However, the effect of melatonin administration as the scavenger of free radicals during estrus on reproductive efficiency and embryo production has limited attention in animals.

To improve the superovulatory response in Thai Holstein crossbreeds raised under high ambient temperature (21–23). It is important to decrease the adverse effect of heat stress on superovulatory responses. The purpose of the present study was to develop superovulation protocols for Thai-Holstein crossbreeds under heat stress conditions by examining the effect of heat stress with melatonin treatment on the superovulatory responses and embryo characteristics in Thai-Holstein crossbreeds under heat stress conditions.

## Materials and Methods

### Animals

Cycling non-lactating Thai-Holstein crossbreeds were used in the experiment. All cows had good body condition scores ranging between 3 and 3.5 (1–5 scale) and were recorded of the body weight using cow weighing tape. The research proposal of this project was approved by the Institutional Animal Care and Use Committee based on the Ethics of Animal Experimentation of the National Research Council of Thailand [Reference No. 660201.2.11/532 (116)].

### Chemical and Melatonin

Unless otherwise stated, all chemicals used in the present study were purchased from Sigma-Aldrich Chemical Company (St. Louis, MO, USA). Melatonin solution was prepared by dissolving in 2.5 % ethyl alcohol as described previously (24).

### Estrus Synchronization, Superovulation, and Artificial Insemination

**Figure 1:**
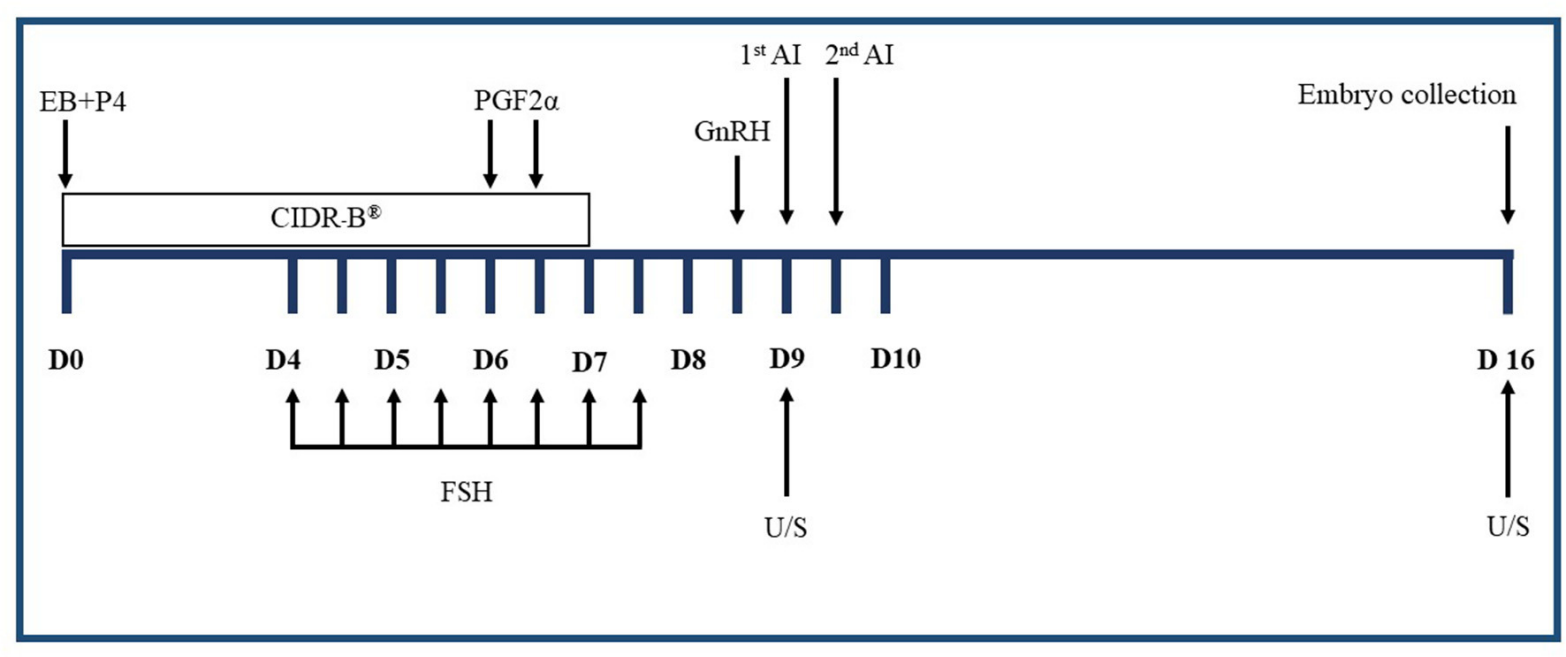
Superovulation treatments in the experiment. CIDR-B®, intravaginal device impregnated with 1.56 g progesterone; EB, estradiol-17β; P4, progesterone; FSH, follicle-stimulating hormone in eight decreasing doses; PGF_2α_, *prostaglandin* F2α; GnRH, gonadotrophin-releasing hormone; 1^st^ AI, the first artificial insemination; 2^nd^ AI, the second artificial insemination; D, day of superovulation treatment; U/S, ultrasound.

Figure 1 shows the traditional superovulation protocols that induce multiple gonadotropin treatments described previously (25, 26). Briefly, on a random day of the estrus cycle (Day 0), estrus was synchronized using a CIDR-B device (Eazi-Breed CIDR-B®, Zoetis Animal Health, Kalamazoo, MI, USA) and an intramuscular (IM) injection of 5 mg estradiol-17β plus 50 mg progesterone (SRC Animal Health, Pak Chong, Nakhon Ratchasima, Thailand). On Day 4, 400 mg of FSH (Folltropin®-V, Bioniche Animal Health, Belleville, ON, Canada) were given IM twice daily in a decreasing dose over 4 days. On the morning of Day 6, 25 mg of PGF2α (Lutalyze®, Zoetis Animal Health, Kalamazoo, MI, USA) was administered IM and repeated 12 h later. The CIDR-B® was removed on the morning of Day 7. The ovulation was induced using 0.01 mg of GnRH (Receptal®, MSD, Unterschleissheim, Germany) IM in the evening of Day 8. All cows were inseminated twice using frozen semen.

### Ova and Embryo Recovery

At 7 days after insemination (Day 16), ova/embryos were collected using a nonsurgical technique to flush the uterine horns described by Ratsiri et al. (25). The flushing media was Dulbecco's phosphate-buffered saline supplemented with 1% (v/v) fetal calf serum (Biological Industries, Beit Haemek, Israel). The recovered embryos were evaluated and graded according to the criteria of Lindner and Wright (27). Only embryos graded A and B were considered transferable; the others were determined as unfertilized ova and degenerated embryos as in our previous study (28). The percentages of transferable and degenerated embryos were calculated based on fertilized ova/embryos.

### Ultrasound Examination

Transrectal ultrasound was performed twice on Day 9 (before insemination) and Day 16 (before embryo collection) using an ultrasound machine (HS-2000 ultrasound scanner; Honda Electronics Co., Toyohashi, Japan) to record large follicles (≥10 mm), corpora lutea (CL), and unovulated follicles (≥9 mm). Ovarian response was determined by summation of the unovulated follicles to the CL. Ovulation rate was determined by dividing the CL number by the ovarian response (29).

### Blood Sample Collection and Enzyme Analyses

Blood samples were collected from the coccygeal vein using 10-ml vacutainer tubes containing heparin and immediately placed in ice. Blood collections were done thrice at 0 h (Day 8) just before the melatonin treatment, at 24 h after the initiation of the melatonin treatment (after insemination time), and at Day 16 before embryo collection. The plasma was separated by centrifugation (350xg for 20 min at room temperature) and stored at −20 °C until enzyme activity measurements.

### Malondialdehyde (MDA) Determination

MDA concentration, an index of lipid peroxidation in the blood samples, was measured using the thiobarbituric acid (TBA) reaction as performed following the instructions provided by Ratchamak et al. (30). Blood samples were added to 0.25 ml of ferrous sulfate (0.2 mM) and 0.25 mL of ascorbic acid (1 mM), and they were then incubated for 60 min in a 37°C water bath. Next, the samples were added to 1 mL of trichloroacetic acid [15% (w/v)] and 1 mL of TBA [0.375% (w/v)] before boiling for 10 min. The samples were cooled down to 4°C to stop the reaction. Finally, the samples were centrifuged at 800xg for 10 min at 4°C. Supernatants (2 mL) were used for analysis using a UV-Visible spectrophotometer (Analytikjena Model Specord 250 plus) at 532 nm.

### Superoxide Dismutase (SOD) Determination

The activity of the antioxidant enzymes SOD was determined following the instructions described by Mussa et al. (31). Briefly, 10 μL of plasma was mixed with 835 μL of a solution containing cytochrome C (1 mM) and xanthine (50 mM), and 155 μL of xanthine oxidase was diluted in sodium phosphate/EDTA buffer (50 and 100 mM, respectively, pH 7.8). Then the absorbance was determined every 5 min in a spectrophotometer fitted with a temperature regulator maintained at 25°C. The concentration of xanthine oxidase was calculated to generate the optimum amount of O^2−^, with a consequent reduction of cytochrome C that was calculated as the rate of cytochrome C reduction of 0.025 units of absorbance/ min (at a wavelength of 550 nm); the basis of this calculation is that 1 unit of total SOD activity corresponded to 50% of this value. Therefore, SOD activity in the sample decreased the rate of cytochrome reduction compared to the blank.

### Temperature-Humidity Index (THI)

Ambient temperature (temp; °C) and relative humidity (RH; %) in the farm area in each superovulation session (between Day 0 and Day 16 of the treatment) were recorded using an automatic temperature and humidity meter (data logger; EL-USB-2). The mean of THI can be calculated by the following equation (32):


$$\begin{array}{l} \text{THI}=\left(1.8\;\times \text{ temp }+\;32\right)\;-\;\left(0.55\;-\;0.0055\;\times \text{ RH}\right)\\ \;\times \;\left(1.8\;\times \text{ temp }-\;26\right) \end{array}$$

### Experimental Design and Statistical Analysis

The experiment was conducted from April to September, with THI above 77, as previously reported (3). Cows were randomly assigned to one of two treatments (melatonin and control groups) to be superstimulated in a double cross-over design. The superstimulation sessions were carried out four testing periods per cow. All cows received the two treatments at an interval of at least 45 days. To increase the confidence of the experiment, each cow received the same alternate treatment in testing periods 3 and 4. In the melatonin group, cows received 18 mg./50 kg of body weight simultaneously with GnRH injection (33), while cows in the control group received none. The superovulatory responses and ova/embryo recovery were determined. The MDA concentration and activity of SOD enzymes between groups were determined.

All data were examined using the SAS statistical software (34). Data were first tested for normality and homogeneity of variance and then analyzed by PROC ANOVA as a double cross-over design. Using Duncan's new multiple range tests (DUNCAN), treatment groups were compared for differences. The significant differences were considered when *p* < 0.05. The statistical model was as follows:


$$\begin{array}{l} {\text{y}}_{\text{ijκ}}=\text{μ}+{\text{ρ}}_{\text{i}}+{\text{γ}}_{\text{j}}+{\text{τ}}_{\text{κ}}+{\text{ε}}_{\text{ijκ}} \end{array}$$

Where y_ijκ_ = observation values such as ovarian response, number of large follicles, unovulated follicles, number of corpora lutea, ovulation rate, total embryo/ova, transferable embryo, degenerated embryo and fertilized ova on treatment k (k = 1 to 2) at testing period i (i = 1 to 4) and cow j (j = 1 to 6); μ = overall mean; ρ_i_ = the effect of testing period i (i = 1 to 4); γ_j_ = the effect of cow j (j = 1 to 6); τ_κ_ = the effect of treatment k (k = 1 to 2); ε_ijκ_ = the effect of experimental error.

For MDA and SOD analysis, the experiment was carried out as a grouped student “t” test to compare the means by PROC TTEST. Results were presented as the mean ± SE. At a *p* < 0.05, results were considered significantly different within day 0 (before melatonin administration), day 9 (after insemination), and day 16 (before embryo collection). Twelve (12) replications were conducted for parameters.

## Results

The averages temperature and relative humidity during the experiments for four testing periods were 29.84 °C and 70.30%, respectively. The average for THI was 81.06. These results demonstrated that the present study was conducted during severe heat stress.

### The Ovarian Responses After Superstimulation

The ovarian follicle responses are summarized in Table 1. The mean numbers of ovarian responses, large follicles, and corpora lutea were significantly greater in melatonin group than in control group (*p* < 0.01), with non-significant in both unovulated follicles and ovulation rates (*p* > 0.05).

**Table 1:** Effect of melatonin treatment (mean±SE) with the superovulatory responses under heat stress condition.

**Parameters**	**Control** **(*n* = 12)**	**Melatonin** **(*n* = 12)**	***P*-value**
Ovarian response^1^ (n)	14.14 ± 1.57^b^	20.20 ± 0.92^a^	**<0.0001**
Number of Large Follicles (n)	13.86 ± 1.55^b^	19.50 ± 0.98^a^	**<0.0001**
Unovulated Follicles (n)	2.21 ± 0.56	1.70 ± 0.45	0.8498
Number of Corpora Lutea (n)	11.93 ± 1.48^b^	18.50± 0.74^a^	**<0.0001**
Ovulation rate (%)	85.58 ± 1.92	92.15 ± 3.71	0.1373

*Within each row, mean ± standard error (SE) with different superscript differed significantly (p < 0.01: shown in bold)*.

1 *summation of the unovulated follicles to the CL*.

### The Ova/Embryo Recovery After Superstimulation

**Figure 2:**
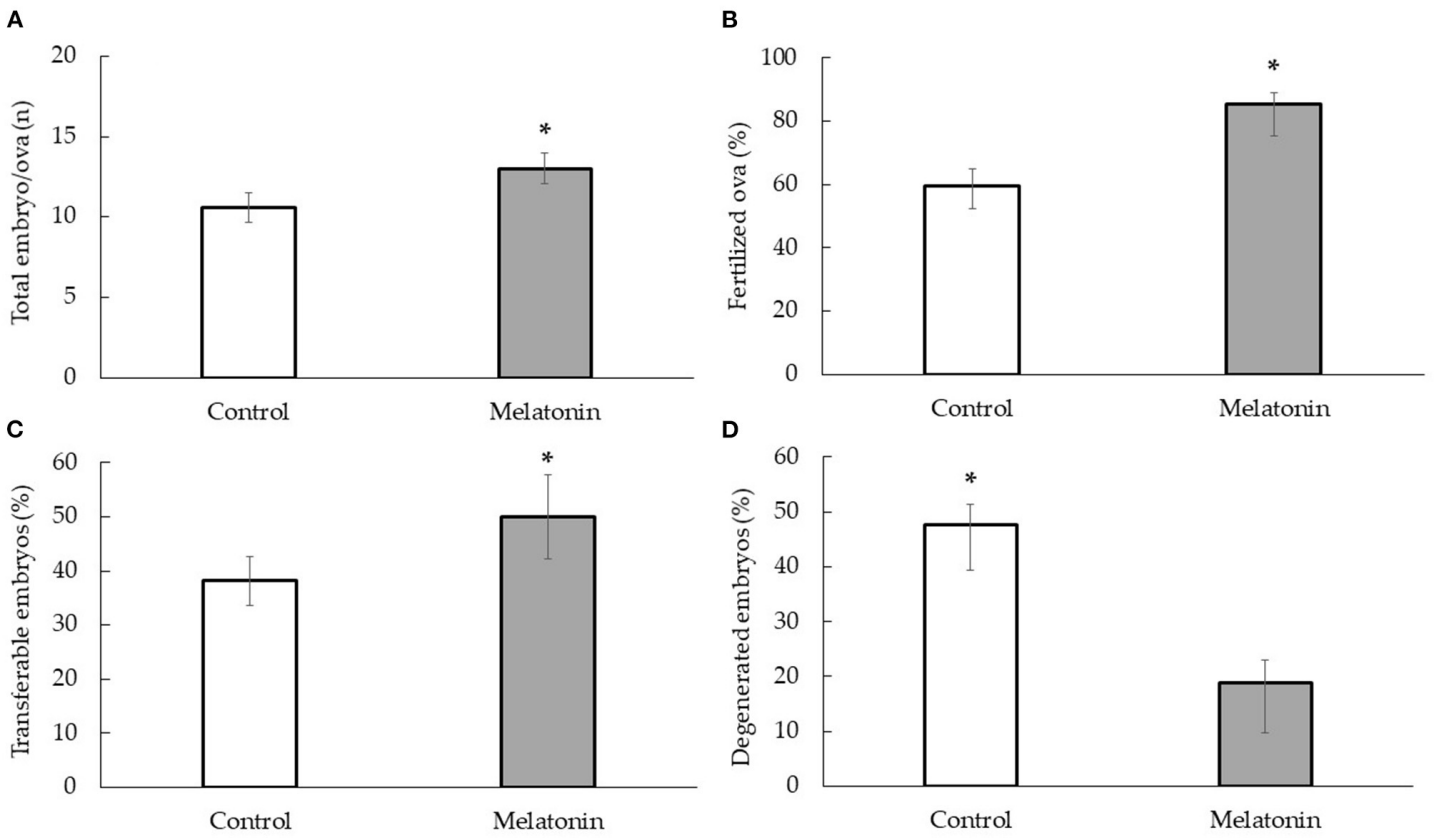
Effect of melatonin treatment with the embryo/ova collection under heat stress condition; total embryo/ova (n) **(A)** fertilized ova (%) **(B)** transferable embryos (%) **(C)** and degenerated embryos (%) **(D)** in Thai-Holstein crossbred cows; * is significant value within treatment (*p* < 0.01).

The results of embryo/ova recovery are shown in Figure 2. The number of total embryo/ova recovery in the melatonin group was significantly higher than that in the control group (13.00 ± 0.92 vs. 10.60 ± 0.96, *p* < 0.01). In addition, the percentage of fertilized ova and transferable embryos was significantly greater in the melatonin group compared with the control group (85.33 ± 4.38% vs. 59.85 ± 6.71% and 50.00 ± 7.85% vs. 38.14 ± 4.43%, *p* < 0.01) which was a significant decrease in a percentage of degenerated embryos (18.90 ± 07% vs. 47.75 ± 3.57%, *p* < 0.01).

### MDA and SOD Levels

As shown in Table 2, before melatonin administration, there had no differences in the activity of the MDA concentrations and antioxidant enzymes SOD (*p* > 0.05). However, MDA levels were significantly greater in the control group on Day 9 and Day 16 of the superovulation program (*p* < 0.05). Meanwhile on those days, the activity of the antioxidant enzymes SOD was higher in the melatonin group than in the control group (*p* < 0.05).

**Table 2:** Effect of melatonin treatment with MDA and SOD under heat stress condition in Thai-Holstein crossbred cows.

**Days of blood collection**	**MDA (μM/mL)**			**SOD (U/mL)**		
	**Control**	**Melatonin**	***P*-value**	**Control**	**Melatonin**	***P*-value**
Day 0	1.33 ± 0.16	1.10 ± 0.15	0.276	11.46 ± 0.69	11.07 ± 0.97	0.6376
Day 9	1.18 ± 0.03^a^	0.79 ± 0.09^b^	**0.0457**	10.88 ± 0.79^b^	14.15 ± 0.45^a^	**0.0499**
Day 16	1.67 ± 0.13^a^	0.96 ± 0.12^b^	**0.0348**	11.56 ± 0.77^b^	14.3 ± 0.57^a^	**0.0057**

*Within each row within parameter, mean ± standard error (SE) with different superscript differed significantly (p < 0.05: shown in bold). Day 0: before melatonin administration. Day 9: after insemination time. Day 16: before embryo collection*.

## Discussion

The results of the present study demonstrated the successful alternative protocol of superovulation with melatonin administration during estrus cycling in Thai-Holstein crossbred cows under heat stress conditions. The melatonin group significantly increased the ovarian responses, large follicles, and corpora lutea. Furthermore, the fertilized ova and transferable embryos were significantly greater, whereas the degenerated embryos significantly decreased with the traditional superovulation protocol. Besides, the control group had a more significant lipid peroxidation; meanwhile, the melatonin groups had a higher concentration of the activity of the antioxidant enzymes SOD.

Superovulation is the process of having the cows produce more follicles than the average count in each estrus by gonadotropin hormone and increasing the number of embryos. However, our previous study (25) reported that a high THI over 80–85 significantly decreased superovulatory efficiency by decreasing ovulation, fertilization, and transferable embryos response in Thai-Holstein crossbreeds. Meanwhile, the degenerated embryos significantly increased. Besides, Ratchamak et al. (3) inferred that the superovulatory response in those cows is apparently affected by heat stress starting at a THI of 72 and becoming severe when THI levels more than 77. Heat stress is an important factor in increasing oxidative stress in oocyte and embryo development (11, 35). Heat stress negatively changed the follicular dynamics, and reduced numbers of large follicles in the follicular wave might be due to the early development of the follicle being heat-sensitive (36). In a study of *in vitro* maturation of bovine oocytes, the direct heat shock at 41°C to immature oocytes for 6–12 h showed reduced maturation and embryo development (37). Together with follicle development, heat stress also influences fertilization and embryo quality outcomes. Sperm incubation at high temperature for 4 h decreased their motility and plasma membrane integrity resulting in increased sperm damage (38). Meanwhile, the early-stage embryos are more heat-sensitive to elevated temperatures than advanced-stage embryos (35, 39, 40). However, the current study indicates that treatment with melatonin effectively increased the number of large follicles, ovarian response, and corpora lutea (Table 1) associated with a more significant number of fertilized ova and transferable embryos while a significantly lower degenerated embryo (Figure 2). According to Song et al. (33), superovulation with melatonin injection in cycling sheep increases transferable embryos (morula and blastocyst). They found higher levels of melatonin in the bloodstream. Therefore, it is presumed that the efficiency of superovulation in melatonin treatment occurred after the administration of melatonin.

Melatonin is a hormone mainly produced by the pineal gland and found in the bloodstream and other fluids, including follicular fluid (41). Melatonin is one of the most widely used as antioxidants; it has mainly two functions as direct and indirect ability to reduce oxidative damage (42). Melatonin scavenges free radicals such as superoxide anion, hydroxyl radical, and hydrogen peroxide, which damage the DNA and lipid of the cell membrane and accelerates cell apoptosis (43). While the indirect actions stimulate the activity of the antioxidant enzymes, including SOD via the membrane receptors of melatonin (44). The beneficial effect of melatonin supplementation as a powerful agent against ROS from oxidative stress and degeneration has been reported during *in vitro* embryo production by supplementing in the culture medium to promote oocyte and embryo development. For instance, melatonin supplementation in the culture medium improves buffaloes' oocyte maturation and embryo yields (15). Melatonin also promoted bovine primordial follicles activation *in vitro* ovarian cortical tissue culture (16).

In the present study, it was essential to clarify whether increasing superovulatory responses (both ovarian responses and embryo quality) under heat stress conditions relate to melatonin's scavenger properties. Therefore, the concentrations of MDA (lipid peroxidation indicator) and the activity of the antioxidant enzymes SOD were evaluated to confirm the activity. Our study shows that MDA decreased while SOD increased in the melatonin group (Table 2). SOD responds to the dismutation of the toxic superoxide radical to hydrogen peroxidase and is considered the first intracellular defense against reactive oxygen species (45). Higher plasma SOD activity in the melatonin group might be due to the physiological upgrading of this enzyme to neutralize superoxide radical challenges (45). Our results implied that melatonin could be a strong antioxidant potential as a scavenger of free radicals by preventing oxidative stress resulting from heat stress; thus, the superovulatory responses were significantly improving with melatonin treatment.

Another critical point that might be considered on the melatonin roles is that melatonin regulates the cycling of reproductive activity. Previous studies demonstrated that melatonin stimulates the GnRH secretion through the hypothalamus-pituitary-gonadal, which affects the secretion of sex hormones on reproductive organs or directly acts on melatonin receptors in the ovaries to regulate sex hormone secretion (46, 47). In mammals, there are two melatonin receptors: melatonin receptor 1 (MT1) and melatonin receptor 2 (48, 49). The release of LH before ovulation was found a high expression of MT1 in the granulosa cells, and the melatonin level was increased in follicle fluid (20). Moreover, melatonin levels might relate to an increase in follicle diameter, where melatonin levels in follicles were higher than in pre-ovulatory blood in humans (50). Ovarian function was directly affected by melatonin, where the accumulation of melatonin in the follicle promotes follicle growth (19).

Therefore, in case of heat stress, that could affect less aromatase activity of granulosa cells and reduce the estradiol concentrations in large follicles (51), together with increased oxidative stress, suggesting that the melatonin combination with FSH in superovulatory protocol could promote more ovarian response.

## Conclusions

In conclusion, we showed that increased activity of the antioxidant enzymes SOD after melatonin administration was associated with decreasing of MDA and improving the ovarian response and embryo quality in superstimulated cows affected by heat stress. The melatonin administration was proper for alternative superovulation techniques to improve the efficiency of superovulatory under tropical climate conditions in the Thai-Holstein crossbreed.

## Data Availability Statement

The raw data supporting the conclusions of this article will be made available by the authors, without undue reservation.

## Ethics Statement

The animal study was reviewed and approved by the Institutional Animal Care and Use Committee based on the Ethics of Animal Experimentation of the National Research Council of Thailand. Written informed consent was obtained from the owners for the participation of their animals in this study.

## Author Contributions

The concept of manuscript was created by RR and VC. The methodology was conducted by RR, PT, and YS and analyzed by RR, WB, and VC. All authors have read and approved to the published version of the manuscript.

## Funding

This work was financial supported by Thailand Research Fund (TRF) under Research and Researcher for Industries (RRI) (PHD62I0032) by Mary Anne Co., Ltd. and the Basic Research Fund of Khon Kaen University.

## Conflict of Interest

The authors declare that the research was conducted in the absence of any commercial or financial relationships that could be construed as a potential conflict of interest.

## Publisher's Note

All claims expressed in this article are solely those of the authors and do not necessarily represent those of their affiliated organizations, or those of the publisher, the editors and the reviewers. Any product that may be evaluated in this article, or claim that may be made by its manufacturer, is not guaranteed or endorsed by the publisher.
